# Trail Use, Motivations, and Environmental Attitudes of 3780 European Mountain Bikers: What Is Sustainable?

**DOI:** 10.3390/ijerph182412971

**Published:** 2021-12-08

**Authors:** Tom Campbell, Lewis Kirkwood, Graeme McLean, Mark Torsius, Geraint Florida-James

**Affiliations:** 1 School of Applied Sciences, Edinburgh Napier University, Edinburgh EH11 4BN, UK; L.Kirkwood@napier.ac.uk (L.K.); G.Florida-James@napier.ac.uk (G.F.-J.); 2 The Mountain Bike Centre of Scotland, Peel Tower, Glentress EH45 8NB, UK; 3 Developing Mountain Biking in Scotland, Peel Tower, Glentress EH45 8BN, UK; graeme.mclean@scottishcycling.org.uk; 4 International Mountain Bike Association, 3843 GD Harderwijk, The Netherlands; Mark.torsius@imba-europe.com

**Keywords:** mountain biking, sustainability, riding preferences, trail-use, trail-maintenance

## Abstract

Background: The extent to which mountain biking impacts upon the environment is largely determined by rider behaviours. The purpose of this study was to gain a better understanding of how mountain bikers interact with the natural environment and explore their attitudes towards sustainability. Methods: 3780 European mountain bikers completed an online cross-sectional survey. Results: Connection to nature was an important source of motivation and the use of mountain bike trails has increased rider’s appreciation of and willingness to protect nature, with a large majority having taken direct action to do so. Mountain bikers are prepared to contribute towards trail maintenance through the provision of labour or financially. Although most mountain bikers make use of wet trails and illegal trails, incidence of conflict is relatively low. A range of characteristics were identified as being fundamental elements of sustainable trails, both in relation to the sustainability of the trail itself and in terms of wider environmental sustainability. Conclusions: European mountain bikers care about the sustainability of the natural environment. Self-reported attitudes and behaviours suggest a willingness to reduce environmental impact and actively protect nature.

## 1. Introduction

Mountain biking is an increasingly popular form of outdoor recreation and adventure tourism [1]. Several disciplines of mountain biking have emerged, and current trends suggest that trail and enduro riding are becoming the most prevalent [2]. These riding styles encompass the use of a wide range of trails, including machine built, hand-dug and wild, or natural, with enduro tending towards more natural, downhill-orientated tracks. All trail-based activities cause a degree of degradation of vegetation and soil, with the effect generally being curvilinear [3] and moderated by an interaction between environmental factors, user type and user behaviour. The impact of mountain biking upon soils and vegetation has generally been shown to be equivalent to that of hikers [4,5,6,7,8] and significantly less than that occurring from equestrian or motorised vehicular use [8,9]. Trails that include high grades and low slope alignments are associated with greater damage [6,9,10], as is inadequate drainage or being located within wet areas [9,11,12,13]. These characteristics are more likely to be found on unauthorised trails (i.e., trails that have been constructed without consent), which may also be described as illegal or unofficial, without increasing their susceptibility to degradation [14]. While use of unauthorised trails is theoretically controlled by local land access rights, which differ markedly across Europe [15,16], most mountain bikers prefer to ride on narrow trails (known as single track) in natural areas [15,16,17,18] and a preference for unauthorised trails has previously been reported [19]. These preferences may potentially increase damage to vegetation and soils, although there is currently a lack of quality evidence [20].

The notion that mountain bikers cause disproportionate environmental damage persists [21,22] and may be partly explained by social conflict, understood to arise where fundamentally different beliefs, values and norms are held by alternative types of recreationists [23]. Social conflict among trail users is a prevailing issue; for example, it was recently reported that approximately half of mountain bikers in Slovenia had encountered conflict with other users over a 12-month period [16]. Clearly, use of natural or shared trails increases the likelihood of different user groups coinciding, fuelling the potential for social and interpersonal conflict. Objections levelled at mountain bikers range from factors inherent in the use of a bicycle, such as noise, speed of movement and the presence of a machine, to behavioural considerations, e.g., riding in large groups, irresponsible or dangerous riding or clothing choice, coupled with a perception of causing damage or being disrespectful to the environment [22,24]. It has even been suggested that mountain bikers could be perceived as deviant, such is the contrast between their use of nature and well-established ideals of environmental engagement [25]. Much of this social conflict appears to originate from the assumption that mountain bikers have different attitudes and motivations for accessing the natural environment than other user groups [21]. However, while hedonic motives, including fun, thrill seeking and risk, undoubtedly drive some mountain bikers [5,15,26,27], affective motives, including appreciation of nature, may actually be more common [5,27]. Yet, relatively little is currently understood about mountain bikers’ attitudes and behaviours in relation to the environment and it is possible that some sources of conflict are unfounded or underpinned by misconceptions about different users’ attitudes and beliefs. There are certainly behavioural indicators to suggest mountain bikers feel a responsibility for, or connection to, the environment. For example, trail maintenance has the potential to mitigate the impacts of mountain biking upon the environment and trail advocacy groups and trail builders’ associations are becoming more commonplace, with volunteer numbers growing at pace. Participation in the “Take Care of Your Trails Initiative” that originated in Scotland in 2015, and is now Europe wide, doubled between 2017 and 2019, when over 2500 volunteers provided 15,000 volunteer hours over hundreds of organised dig days [28]. There is also some evidence that environmental concerns are the biggest motivation for participation in trail maintenance activities, at least amongst American trail builders [29]. 

The aim of the current study was to gain an empirical understanding of trail preferences, motivations, environmental attitudes and behaviours of European mountain bikers. Establishing not only where mountain bikers choose to ride, but why and when, is important for planning, land management and maintenance, while understanding environmental attitudes and behaviours may have implications for improving the sustainability of the sport. 

## 2. Materials and Methods

This was a cross-sectional study exploring the characteristics, riding habits, trail preferences, attitudes and behaviours of European mountain bikers. As no standardized measure is available to capture this information, an online survey tool was developed using a sequential exploratory strategy. Construct development followed a three-stage process, which involved identifying the key themes within the literature, acknowledging discrepancies and finally ordering themes into categories. The content and constructs of the developed questionnaire were validated by consensus by sector experts from the International Mountain Bike Association, BikePlan Switzerland, Danish Gymnastics and Sports Associations, International Mountain Bike Association, Município de Águeda, Scottish Cycling Union, The Mountain Bike Centre of Scotland, Mountain Bike Information Office and Fagskolen Tinius Olsen. The survey was initially developed in English and subsequently translated into German, Italian and Portuguese language versions. Following a pilot, minor amendments were made to reflect national differences in terminology. The survey was distributed using Qualtrics software and was open between March and July 2020. The inclusion criteria required respondents to be a mountain biker aged 16 or over and ordinarily resident within a European country. Participants were initially presented with an information sheet and were required to provide informed consent by ticking a check box before accessing the survey. This study was approved by the School of Applied Sciences Research Integrity Committee, Edinburgh Napier University (#SAS0075).

### 2.1. Survey Tool

The survey tool comprised five sections: demographics and rider characteristics, trail use and preferences, motivations for mountain bike participation, attitudes towards sustainable trails and environmental behaviours. There is no universally applied approach to the classification of different types of mountain bikers but, previously, consideration has been given to riding discipline, or style, trail preference or bike type. We included measures of both trail preference and riding discipline in the present study. Preference for trail type was measured with a series of questions relating to both the frequency of use and reason for using different types of trail while discipline was measured using a single questionnaire item. The trail typologies and disciplines included within the study are shown in Table 1. The survey contained 42 questions in total, with all but one requiring categorical or ordinal responses to be provided by means of drop-down menus, Likert scales and “select all that apply” options; for example: “Thinking about your own use of trails and personal attitude towards nature, please indicate to what extent the following statements apply to you: My use of and access to trails has led me to change my behaviours to reduce my environmental impact—strongly agree, agree, disagree, strongly disagree”. The final item required participants to provide a free text response to the question, “What do you consider to be a sustainable trail?”. 

### 2.2. Data Analysis

Quantitative data were analysed using Statistical Package for Social Sciences version IBM SPSS Statistics 26 (IBM Inc., Chicago, IL, USA). Chi-squared tests of independence (Bonferroni adjusted) were performed to investigate differences in self-reported behaviours and attitudes according to country of residence. A thematic analysis of all free text responses to the open question, “What do you consider to be a sustainable trail”, was performed in NvivoPro12 (QSR International, London UK), with non-English language responses being translated by native speakers prior to analysis. 

## 3. Results

### 3.1. Study Population Characteristics and Mountain Bike Experience 

Only mountain bikers aged 16 and above and ordinarily resident within Europe were included in the study. The survey was accessed by 4500 individuals and a total of 3780 participants provided consent and complete responses (Table 2). The majority of respondents were male (60%) and most believed themselves to be intermediate (50.2%) or expert-level (43.2%) riders, with relatively few beginners (3.7%) or professionals (2.9%) completing the survey. Over half of respondents were aged between 26 and 45 years, while fewer than 10% of respondents were under 26 years of age. Most respondents had been mountain biking for over a decade (52.9%) and over a third were involved in competitive mountain biking, most commonly at a regional level (36.1%) although a quarter competed nationally (25.1%), and nearly 10% internationally. In total, 29 European countries were represented in the sample and data from those associated with medium or high response rates (n ≥ 100) were presented separately.

### 3.2. Participation in Mountain Biking Disciplines and Motivations for Riding

European mountain bikers participate in a range of mountain biking disciplines, with trail (31.2%) and enduro (25.7%) being the most popular (Table 3). Associations were found between country and participation. Denmark and the Netherlands reported significantly higher involvement in cross-country riding (X2 = 151.83, df = 7, *p*< 0.001) and significantly lower rates of enduro riding (X2 = 153.47, df = 7, *p* < 0.001) than any other country while freeride and downhill were more popular in Switzerland, Norway and the UK than in Netherlands, France and Denmark (X2 = 68.53, df = 7, *p* < 0.01).

While various motivations for riding were reported, exercise/health (20.2%) was most widely cited, closely followed by connection to nature (19.2%). Play (17.4%) was the third most popular reason for participation in mountain biking with accomplishment (1.3%) being the least frequently reported source of motivation. Riders in Norway, Denmark and the UK reported lower motivation towards a connection to nature compared with all other countries (X2 = 132.50, df = 7, *p* < 0.001), and exercise was a less frequently reported motivation for riders in Switzerland and Germany than in the Netherlands, Denmark, Norway or the UK (X2 = 105.82, df = 7, *p* < 0.001). Although risk was not reported as a common motivation for European mountain bikers (1.3% to 5.5%), riders in the UK were more likely to be motivated by risk than any other riders (X2 = 74.51, df = 7, *p* < 0.001).

### 3.3. Trail Use and Preference

**Figure 1:**
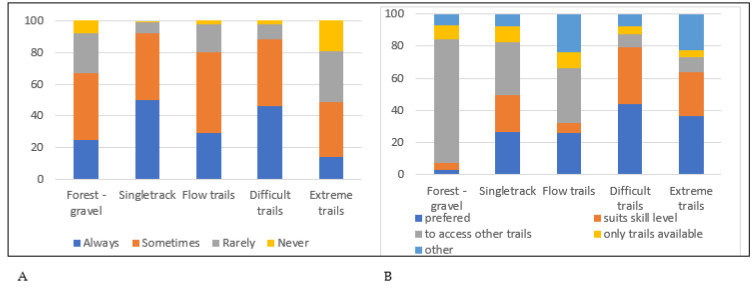
Self-reported use of different trail typologies: (**A**) frequency of trail use; (**B**) reason for trail use.

Singletrack was the most frequently used trail type, with over 90% of riders “always” or “sometimes” riding singletrack, followed by difficult trails, flow trails, forest or gravel (Figure 1). Although extreme trails were the least frequently used, in relative terms, nearly half of the surveyed riders reported use of this type of trail. However, there is a clear disparity between use of, and preference for, certain trail types. For example, over three quarters of riders using forest roads or gravel tracks (77.4%), and a third using singletrack (33%) or flow (34%) trails, claim to do so only as a means of accessing other trails. Conversely, a large proportion of riders using difficult and extreme trails do so through preference and alignment with their skill level (79.2% and 64.1%, respectively). 

### 3.4. Attitudes towards Trail Access

**Figure 2:**
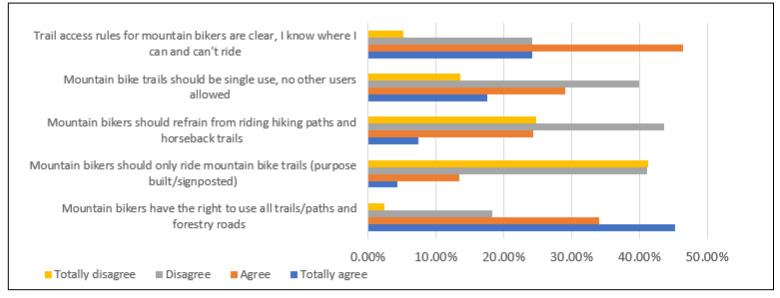
Attitudes towards trails access.

Most mountain bikers (70.6%) claim that they are clear about where they are permitted to ride. While just under half (46.5%) believe that mountain bike trails should be reserved for bikers only, fewer than one in five reported that mountain bikers should only use bike-specific trails. Indeed, more than 80% of riders believe mountain bike riders should be able to use all trails/paths and forestry roads, including hiking paths and horseback trails (Figure 2).

### 3.5. Unauthorised Trail-Use and Social Conflict

Across the whole sample, a majority of respondents report that they use unauthorised trails, with 21.3% doing so occasionally and 36.7% often (Table 4). There was an association between country of residence and reported use of unauthorised trails (X2 = 482.28, df = 21, *p* < 0.001). Danish riders report the lowest regular use of these trails while German riders are significantly more likely than any others to use unauthorised trails and to report a lack of authorised trails as a reason for doing so (X2 = 217.39, df = 7, *p* < 0.001).

**Figure 3:**
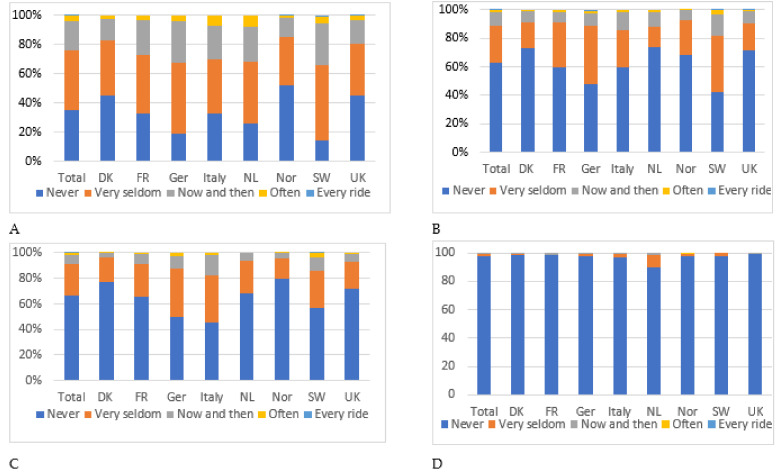
Responses to the question, “How often have the following scenarios occurred when riding unofficial trails?”. (**A**) Other users make negative comments. (**B**) Other users block the trail and have a discussion with me. (**C**) Discussion with landowner or manager. (**D**) Issued a fine/penalty.

The majority of respondents have experienced social conflict at some point while riding unauthorised trails (Figure 3), and the most common form of conflict involves other users making negative comments (64.5%). This remains a relatively infrequent occurrence, however, with over 60% having experienced this either “very infrequently” or “now and again”. Riders in Switzerland reported the highest incidence of this form of conflict and significantly more than in Norway, Italy, France, Denmark and the UK (X2 = 173.31, df = 21, *p* < 0.001). Confrontation with other trail users, e.g., blocking the trail and engaging in a discussion, was the second most common form of conflict, being encountered by 37.6% of respondents. Again, this occurs very infrequently, with fewer than 2% reporting that this happens “often” or “every ride”. Mountain bikers in Switzerland are again more likely than any other riders to have experienced this type of conflict (X2 = 130.83, df = 21, *p* < 0.001). One a third of European mountain bikers have been involved in a discussion with a landowner or manager at some point although this is most likely to have been experienced by riders in Italy (X2 = 122.12, df = 21, *p* < 0.001). 

### 3.6. Environmental Attitudes and Behaviours

Most European mountain bikers (89.4%) report that they ride on wet trails, with nearly one in five believing they have no option to avoid wet trails and 10.8% claiming to enjoy riding in the wet (Table 5). Of those who avoid riding on wet trails just over half claim this is borne out of a desire to prevent damage while the remainder have a dislike of riding wet trails. Associations were found between country and use of wet trails (X2 = 384.43, df = 28, *p* < 0.001), with significantly more riders based in the UK having no option but to use wet trails compared with all other countries. There also was a higher proportion of riders in the UK who ride wet trails for enjoyment than in Switzerland, Norway and Germany.

**Figure 4:**
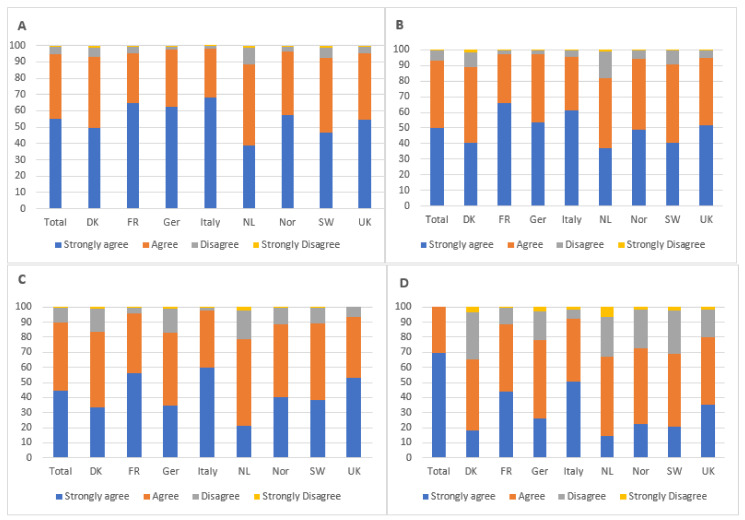
Respondents’ perception of the extent to which their use of mountain bike trails has influenced their attitudes towards nature. (**A**) Increased appreciation of nature. (**B**) Increased willingness to protect trails. (**C**) Taken action to protect nature. (**D**) Changed behaviour to reduce environmental impact.

A substantial majority of European mountain bikers believed that their use of mountain bike trails has increased their appreciation of, and willingness to protect, nature (90%). A similar proportion of riders claim to have taken direct action to protect nature as a result of their use of mountain bike trails. Three quarters of respondents report that they have changed their behaviour to reduce their environmental impact as a consequence of their participation in mountain biking (Figure 4). Associations were found between country and appreciation of nature (X2 = 45.61, df = 21, *p* < 0.01), willingness to protect nature (X2 = 80.80, df = 21, *p* < 0.001), direct action (X2 = 115.22, df = 21 *p* < 0.001) and behaviour change (X2 = 145.42, df = 21, *p* < 0.01) as a result of using mountain bike trails. However, these tended to indicate the strength of feeling rather than the direction. 

**Figure 5:**
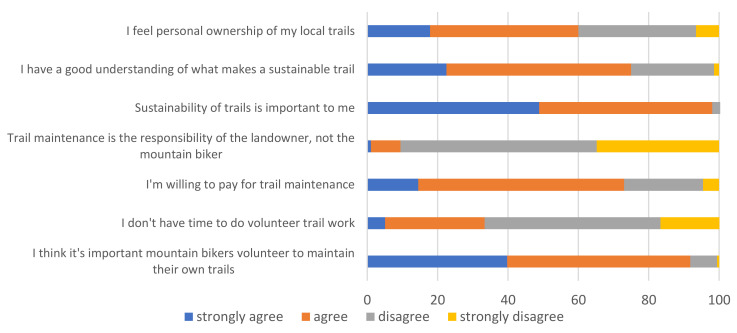
Attitudes towards trail maintenance and sustainability.

Almost all of the survey respondents reported that sustainability of mountain bike trails is important to them (98%), and three quarters (75%) believe that they have a good understanding of what makes a sustainable mountain bike trail (Figure 5). Additionally, most mountain bikers feel a sense of personal ownership of their local trails (60%), with very few (10%) believing trail maintenance to be the responsibility of the landowner. Similarly, a large majority believe it is important to contribute towards trail maintenance, and 91% believe that mountain bikers should volunteer to maintain trails themselves. However, one third claim that they do not personally have enough time to participate in trail maintenance, but most (73%) would be willing to pay for trail maintenance.

### 3.7. Perceptions of Sustainable Trail Characteristics

A total of 1552 respondents provided a free text response to the open question, “What do you consider to be a sustainable trail?”. Two higher-level themes emerged relating to the sustainability of the trail itself or to a broader conceptualisation of environmental sustainability (Table 6). Within the trail-focussed theme, five categories were formed: durability, construction, maintenance, user experience and design features, and a total of 18 codes were split across these categories. Within the theme of wider sustainability, twenty codes were assigned across four main categories: environment and nature, landscape and terrain, facilities and socio-economic factors. Although responses relating to the sustainability of the mountain bike trail itself were the most frequent (n = 1555), wider environmental factors were also widely cited as being characteristics of a sustainable mountain bike trail (n = 1060). Indeed, the single most common category of sustainability considerations was environment and nature (n = 720). The most frequent codes within this category were minimising impact on the wider environment, reducing erosion, preventing damage to flora and fauna, and protecting wildlife. The lower-order theme, landscape and terrain, involved integrating the trail within the landscape, making use of the landscape, or ensuring that there was a minimal visual impact upon the landscape. Within the socio-economic category, minimising impact on other users was the predominant consideration.

Factors relating to the construction process were mentioned by 618 respondents, with drainage and water management being most frequently cited as a fundamental characteristic of a sustainable mountain bike trail (n = 244) followed by use of local (n = 139) or natural (n = 115) materials. Although a relatively high number of respondents mentioned trail maintenance (n = 242) there were disparate views on how this should be operationalised, with approximately half (52%) of responses preferring “low maintenance”, 30% prioritising regular maintenance and the remainder believing “ease of maintenance” to be most important. Respondents felt that a mountain bike trails should be designed to moderate rider behaviours, specifically by ensuring riders keep to the trail therefore minimising trail creep, and by controlling speed and braking.

## 4. Discussion

Firstly, concerning the sample, most respondents were male, with a majority aged between 26 and 45 years old—a demographic profile that aligns with previous surveys and reports [2,16,27,30,31,32] and is therefore assumed to be broadly representative of the wider population of mountain bikers. Similarly, the reported preference for enduro and trail-type riding is also in keeping with worldwide trends [2], although European mountain bikers prioritise trail riding over enduro. While subdisciplines can be differentiated by the nature of the terrain ridden and the characteristics of the bike itself, beyond the competitive environment this delineation is largely arbitrary as riders tend to participate in multiple types of riding [15,16,33,34]. Indeed, in the present study, riding disciplines appear to be interchangeable to a degree, or at least not exclusive, with almost all respondents participating in multiple disciplines.

In terms of trail use, singletrack was by far the most widely used trail type with extreme trails being used the least. Whilst acknowledging that trail classifications are somewhat subjective, this might be construed to suggest that singletrack has the broadest appeal to mountain bikers. However, frequency of use of a given trail type does not necessarily correspond to preference. Indeed, the most reported reason for using singletrack and forest track was as a means of accessing alternative, preferred trail types. This has implications for trail planning and decision making, as well as for future research design as usage should not be conflated with preference. 

Turning to attitudes and behaviour, most European mountain bikers believe that they should be able to ride on any trail but also that mountain bikers should have exclusive use of certain trails which appears somewhat inequitable. Without presuming to understand why respondents have taken this position, there is an argument to be made that there are safety implications for certain mountain bike trails, especially downhill and jump-oriented tracks where other users might pose a hazard. Furthermore, most riders admit to using illegal or unauthorised trails, extending previous findings from a cohort of Slovenian mountain bikers [16], although there were some differences in frequency and rationale for using illegal trails across the different countries. Perhaps unsurprisingly, use of unauthorised trails is greatest in countries where riders report a shortage of appropriate legal trails. Notwithstanding the issue of varied access rights, several earlier studies have shown that mountain bikes on non-designated trails is a significant source of social conflict [35].

Following the lack of authorised trails offerings, the next most common reason for riding unauthorised trails was a belief that doing so is harmless if done during quiet times. Yet, the prevalence of social conflict on unauthorised trails was relatively high, with a vast majority of respondents having encountered conflict at some point. This belief may be better explained by considering the frequency of conflict, which was relatively low. Additionally, although no measure of incidence was included, the sample generally had several years of experience of mountain biking, which might provide further explanation. Nevertheless, it has previously been shown that even where social conflict between mountain bikers and other users is rare, occasional unpleasant encounters have the potential to significantly influence future experiences, and walkers have reported being fearful of further incidents [36]. There may therefore be a difference between perception and reality, with mountain bikers seeking to justify their behaviour based on their own perspective of what constitutes harm.

Interestingly, however, the motivations for participating in mountain biking were found to be similar to those of other groups of outdoor enthusiasts, with exercise and health and connection to nature being the most commonly reported primary motives. Conversely, risk was not found to be a particularly common dominant source of motivation in the sample, despite mountain biking being widely considered an extreme, or adrenaline, sport [37]. This may to some extent reflect the nature of the cohort, as the enjoyment of risk is known to be greater amongst downhill riders [27] who were somewhat underrepresented. Nevertheless, enduro mountain biking is predominantly a gravity-focussed discipline, with rates of injury comparable to downhill mountain biking [38]. It is plausible that any enjoyment of risk or thrill seeking is secondary to the pleasure derived from being in nature for most mountain bikers.

The current study also found that participation in mountain biking increases the perceived appreciation of, and willingness to protect, nature, adding to the evidence that mountain bikers have positive attitudes towards the natural environment [27,39]. Furthermore, this appears to translate into behaviour, with a substantial majority of mountain bikers claiming to have taken direct action to protect nature, and three quarters having changed behaviours to reduce their environmental impact.

The thematic analysis revealed that European mountain bikers appear to have a good understanding of the elements that contribute to the sustainability of mountain bike trails, reporting a varied range of factors relating to both the trail itself and also wider environmental and social sustainability. Interestingly, environment and nature emerged as the most common higher-order themes, again pointing to a sense of environmental responsibility extending beyond merely protecting mountain bike trails. European mountain bikers are also knowledgeable about important elements of sustainable trail design as drainage was identified as the single biggest characteristic of a sustainable trail. Keeping water off the track has certainly been shown to be one of the most important measures for reducing the impacts of mountain biking [12]. Despite this, relatively few riders avoid riding in the wet, which may accelerate the process of erosion [9,11,13]. The proportion of riders using wet trails varied by location and almost certainly reflects localised climactic conditions and regulations governing trail use. When viewed in isolation to the relatively high proportion of riders who continue to ride in wet conditions, suggests that some behaviours may not be entirely cognisant of sustainability or fully align with reported environmental attitudes. This may reflect an inherent contradiction between the desire to enjoy and protect nature and participating in mountain biking for hedonic reasons; however, environmental sustainability is a more complex and nuanced issue. In line with previous findings [16], the current study also shows that European mountain bikers are prepared to contribute to the maintenance of trails, either financially or through voluntary participation. Various mechanisms exist to effectively harness this potential social and financial capital, including pay-to-ride fees, lift tickets, cycling memberships, car parking fees, dig days and trail associations. The latter are generally charitable organisations and often enable mountain bikers to contribute to the upkeep of local trails in the way that they choose, for example through memberships, volunteering, subscriptions and one-off donations; e.g., the Tweed Valley Trail Association [40]. Future research might seek to establish new or optimal models for capitalising on the apparent willingness of mountain bikers to maintain their trails and protect the environment.

This study provides valuable insight into the behaviours and attitudes of European mountain bikers but is not without limitations. The psychometric properties of the survey tool have not been established and there was an overrepresentation of experienced and competent mountain bikers in the sample. The terms illegal and unauthorised trail might have subtly different interpretations across Europe, in part due to legislation governing access rights. There was no consideration for the type of trails being ridden in the wet, which may moderate the extent of any damage. Similarly, electric mountain bikes may have different implications for environmental sustainability, but no measure of e-bike use was included in the survey although e-mountain bikes are believed to represent approximately a third of the current market in large areas of Europe [41].

## 5. Conclusions

European mountain bikers care about the sustainability of the natural environment, and their attitudes and self-reported behaviours suggest a willingness to reduce their impact and actively protect nature. In particular, mountain bikers feel a responsibility for maintaining the trail network. There is an opportunity for national governing bodies and relevant stakeholders to capitalise on this goodwill by establishing mechanisms for mountain bikers to engage with trail maintenance either through monetary support or volunteering with trail associations. Similarly, the possibilities to exploit this financial and social capital towards broader environmental concerns should be explored and may also prove a worthwhile avenue for future research.

The reported preferences for riding illegal or unauthorised trails, allied with relatively high use in wet weather, may lead to higher levels of localised damage. However, due to a lack of systematic evidence, the environmental implication of this behaviour remains unclear. Further, research should be undertaken to measure any environmental impact, especially when aligned with appropriately structured maintenance and repair. Creating separate designated trails for mountain bikers would reduce the incidence of conflict with other user groups, and if not possible, meaning shared trails are to be used, then a level of co-education of trail users needs to be instigated. Lastly, European mountain biking continues to be heavily male dominated; hence, organisations involved in mountain biking should endeavour to engage underrepresented groups to improve the diversity of the sport.

## Figures and Tables

**Table 1:** Categorisation of trail typologies included in the survey.

Trail/Discipline	Characteristics
Forest or gravel	Forest gravel road wider than 2 metres, or double track
Singletrack (easy)	Winding around obstacles such as trees and large rocks
Flowy	Minimal pedalling and braking, rolling terrain, predictable surfaces
Difficult	Natural obstacles and technical trail features
Extreme	Difficult, gravity orientated trails with jumps and unavoidable obstacles
Cross Country (XC)	Variety of off-road terrain combining long climbs and flowing descents
Trail	Like XC but with greater obstacles and more rugged terrain
Enduro	Riding downhill trails at high speed with uphill transitions
Freeride/Downhill	Extremely challenging, jumps, large features, steep gradients
Dirt jump	Riding shaped mounds of dirt/soil to become airborne and perform tricks
Pump track	A circuit of rollers, banked turns and features ridden without pedalling

**Table 2:** Respondent characteristics and response rates by country (n = 3780).

			N	%			N	%
Characteristics	Gender	Female	613	16	Experience	<2 years	251	6.7
		Male	2257	60		2–5 years	690	18.4
		Undisclosed	910	24		5–10 years	828	22.1
	Age	16–18	107	2.8		>10 years	1986	52.9
		19–25	267	7.1				
		26–35	965	25.6	Competes	Yes	1339	35.7
		36–45	1274	33.8		No	2414	64.3
		46–55	867	23.0	Level	Local	568	28.7
		>55	293	7.8		Regional	713	36.1
	Riding Level	Beginner	140	3.7		National	507	25.7
		Intermediate	1886	50.2		International	188	9.5
		Expert	1624	43.2				
		Professional	108	2.9				
	High (>300)	Denmark, Italy, Norway, Switzerland, United Kingdom			Medium (100–300)	France, Germany, The Netherlands		
	Low (25–99)	Austria, Belgium, Bulgaria, Finland, Portugal, Spain, Sweden, Ireland			Very low (<25)	Albania, Andorra, Belarus, Czech Republic, Greece, Hungary, Iceland, Luxembourg, North Macedonia, Poland, Romania, Slovakia, Slovenia		

**Table 3:** Participation in mountain bike disciplines and the motivation for riding (n = 2050).

		Total	DK	FR	DE	IT	NL	NO	CH	UK
Discipline	XC	15.1	25.1	14.8	11.3	13.8	35.7	11.9	7.9	11.4
	Trail	31.2	35.2	31.0	33.0	26.0	28.9	33.1	26.3	31.8
	Enduro	25.7	16.2	33.2	29.4	33.5	15.7	25.0	31.2	25.7
	Freeride/ DH	12.9	8.3	8.4	14.5	15.2	6.4	14.8	17.8	13.0
	Dirt jump	2.8	2.9	0.8	1.70	2.3	1.7	2.5	2.5	4.4
	Pump track	8.2	8.3	6.4	6.79	6.8	6.8	8.9	11.5	8.1
	Miscellaneous	4.1	3.1	4.9	2.83	1.7	3.4	3.6	1.8	3.8
	Connected to nature	19.2	18.8	22.6	22.9	23.7	19.6	17.9	22.6	15.2
	Escape/solitude	16.7	16.2	17.2	20.5	11.4	17.8	15.8	18.6	17.8
	Challenge	17.3	16.3	13.9	20.2	13.7	17.3	17.7	19.5	17.8
	Risk	3.5	2.5	3.3	2.1	4.3	1.3	3.3	2.7	5.5
	Play	17.4	18.1	16.9	12.8	18.9	16.9	19.8	15.5	17.5
	Exercise/Health	20.2	22.6	19.8	17.1	20.7	22.0	21.6	16.4	19.9
	Accomplishment	1.3	1.2	0.8	1.2	1.1	0.4	1.1	1.1	1.6
	Culture	2.2	1.6	4.0	1.5	3.8	2.4	1.3	1.1	2.3
	Other	2.2	2.7	1.5	1.7	2.5	2.2	1.3	2.5	2.5

DK: Denmark; FR: France; DE: Germany; IT: Italy; NL: Netherlands; NO: Norway; CH: Switzerland; UK: United Kingdom.

**Table 4:** Frequency and rationale for riding unauthorised trails (n = 3255).

	Total	DK	FR	DE	IT	NL	NO	CH	UK
**Frequency**									
Never	26.3	44.9	15.6	7.7	27.1	31.3	39.5	12.69	19.9
Occasionally	36.7	42.1	50.3	34.1	35.8	50.0	24.4	52.79	31.6
Often	21.3	5.8	19.1	53.6	14.2	16.6	3.6	29.44	30.8
Unsure	15.7	7.2	15.0	4.6	22.9	2.1	32.5	5.08	17.7
**Rationale**									
Insufficient legal trails	25.7	17.4	19.5	35.8	29.3	19.4	11.9	28.5	24.4
Legal trails unappealing	15.5	10.4	17.0	21.5	12.7	22.2	3.5	20.0	16.1
Convenience	2.1	3.1	3.3	0.6	1.2	0.7	3.8	1.5	2.5
Freedom/Adventure	18.2	17.6	24.9	14.0	19.5	18.1	22.1	15.8	20.4
It is harmless…*	24.8	30.9	24.2	23.1	24.3	27.1	31.8	27.6	20.4
Other	13.64	20.7	11.2	5.0	13.0	12.5	26.9	6.3	16.3

DK = Denmark; FR = France; DE = Germany; IT = Italy; NL = Netherlands; NO = Norway; CH = Switzerland; UK = United Kingdom * “It is harmless if done at quiet times”.

**Table 5:** Mountain bikers’ use of wet trails according to country (%).

	Total	DK	FR	DE	IT	NL	NO	CH	UK
No prevent damage	5.8	5.2	7.7	7.8	9.5	6.3	4.9	9.3	2.3
No, dislike riding in wet	4.8	7.5	4.2	4.7	11.9	5.3	4.5	4.0	1.4
Sometimes	61.0	68.5	63.1	74.0	60.0	66.3	73.0	65.8	38.5
For enjoyment	10.8	5.4	7.1	2.6	1.4	4.2	8.5	3.7	29.8
Yes, as no option	17.8	13.3	17.9	10.9	17.2	17.9	9.1	17.2	27.9

**Table 6:** Content analysis of the question, “What do you consider to be a sustainable trail?” (n = 1555).

**Trail Focussed Sustainability**		
**Category**	**Codes**	**Frequency**
Durability (n = 479)	All weather	105
	Long lasting	105
	Withstands traffic volume	97
	Durable/robust/armoured surface	61
	Resistant to damage	57
	Well built	53
Construction (n = 618)	Natural materials	139
	Local Materials	115
	No/Limited machine use	70
	Natural features	47
	Drainage	244
Maintenance (n = 242)	Low maintenance	126
	Well or regular maintenance	74
	Easily maintained	29
User experience (n = 93)	Sensible gradient	47
	Fun	18
	Safe	16
	Flow	8
	Multiuse	4
Design Features (n = 123)	Minimises trail creep/keeps users on the trail	69
	Speed and braking control	36
**Wider Sustainability**		
**Category**	**Codes**	**Frequency**
Environment and nature (n = 720)	Minimises Impact on wider environment	189
	Reduces erosion	170
	Prevents damage to flora and fauna	147
	Protects wildlife	85
	Minimal digging or removal of trees/roots.	77
	Avoids sensitive and wet areas	46
	Returns to nature after use	6
Landscape and terrain (n = 159)	Integration with nature and landscape	83
	Sympathetic to/makes use of landscape or terrain	55
	Minimal impact on landscape	21
Facilities (n = 90)	Cleaning no litter	21
	Ease of access	34
	No motorised vehicles	17
	Signage	18
Socio-economic (n = 91)	Minimal impact on other users	38
	Economically sustainable or beneficial	15
	Benefits to local community	13
	Designed, built, or maintained in collaboration with community/stakeholders	25

## Data Availability

The data presented in this study are available on request from the corresponding author.
